# Preventing Paraffin-Related Injury

**DOI:** 10.5249/jivr.v1i1.1

**Published:** 2009-07

**Authors:** David C. Schwebel, Dehran Swart

**Affiliations:** ^*a*^Department of Psychology, University of Alabama at Birmingham, USA.; ^*b*^The Paraffin Safety Association of Southern Africa, Cape Town, South Africa.

## Abstract

Paraffin (called kerosene in North America and other parts of the world) is the most commonly used fuel in ‎non-electrified dwellings worldwide. It is especially popular in Africa and South Asia. Although paraffin ‎offers many advantages-especially its comparatively low cost to produce-it poses two major risks of ‎injury. First, paraffin poisoning is common, either through ingestion or through inhalation of smoke and ‎fumes. Second, paraffin is highly flammable, and poses fire risk through multiple causes. This commentary ‎discusses strategies to prevent paraffin-related injury. Prevention of paraffin-related injury must be through ‎multiple strategies, and should include policy-oriented change, changes to the safety of home environments, ‎and behavioral changes targeting how individuals store and use paraffin and paraffin appliances. We review ‎successful prevention strategies in each of these domains and discuss appropriate research and community ‎initiatives that should be implemented to improve paraffin safety among at-risk populations.

## Introduction

Prior to electrification, low-income residents of low- and middle-income nations rely on biomass and fossil fuels to heat their dwellings, cook their food, and light their environment. Among the most popular fuels for these purposes is paraffin (called kerosene in North America and other parts of the world).^1^

A hydrocarbon fuel created through distillation of petroleum, paraffin is invaluable for many reasons. It is relatively inexpensive to produce and consume, readily accessible, and it offers heat, light, and cooking opportunities to millions of individuals who would otherwise be without a domestic source of energy. Paraffin use is documented in most countries of the world, but is used by particularly large numbers of people in Africa and South Asia.

**Risks of Paraffin**

Paraffin poses two major risks of injury, especially to children. The first is through poisoning, either through ingestion or through inhalation of smoke and fumes. Paraffin has the appearance and viscosity of water, so thirsty toddlers sometimes drink it mistakenly. This event is especially problematic in nations like South Africa and Kenya that do not have regulations enforcing safe packaging of paraffin. In those countries, empty beverage bottles are frequently reused to transport and store paraffin. Many households are poorly ventilated, and sub-standard paraffin appliances emit unacceptably toxic chemicals that lead to respiratory problems.

The second risk is burns. Paraffin is highly flammable, and poses fire risk when contaminated by water or other fuels; when used in malfunctioning appliances; when used by youth, intoxicated individuals, or other vulnerable individuals; when used purposely in acts of aggression or self-harm; and in dozens of other situations. Once a fire starts, children are at particular risk of paraffin-related burns due to their reduced mobility, undeveloped risk perception, longer sleeping hours, and greater likelihood to sleep deeply.

**Prevention of Paraffin-Related Injury**

As is the case for most types of injuries, prevention of paraffin-related injury must be multifaceted.^2^ Below, we outline three primary targets for prevention: through policy, through environmental changes, and through behavioral changes. We discuss successes and future directions within each domain.

**Policy Approaches**

Various paraffin-safety initiatives have been put forth by countries worldwide. Australia mandates, for example, that paraffin be dyed blue to prevent confusion with water. Existing evidence raises doubt whether this prevents ingestions or not, partly because young children, too immature to recognize color differences, are the primary poisoning victims.

South Africa, which has one of the world’s largest paraffin-using populations (estimated between 17-20 million individuals who use paraffin daily), has been a leader in policy-initiated approaches to paraffin safety. In 2007, South Africa’s National Minister of Trade and Industry, Minister Mandisi Mpahlwa, declared SANS 1906, a compulsory specification for non-pressurized Paraffin Stoves and Heaters. The standard covered several safety-related topics, including: (a) prevention of fuel leakage and overheating, (b) mechanisms to cause self-extinguishment if the appliance is knocked over, (c) prevention of harmful emissions to the environment, (d) stability and durability of the appliance, (e) prevention of filling the appliance when it is in use, (f) insurance of sustained power output over time, (g) prevention of hot control dials/buttons, and (h) inclusion of pictograms with safety instructions. Legislation like SANS 1906 is a critical linchpin in our battle to prevent paraffin-related injury, but remains completely absent in many nations and deficient in most others.

**Environmental Approaches**

Many aspects of the home and community environment increase risk of paraffin-related injury. Paraffin users worldwide tend to live in low-income dwellings constructed of highly flammable materials such as wood, cardboard, and plastics. Although some are in sparsely-populated rural areas, a large majority live in densely-populated urban settlements where fires can spread quickly and fatally. Some neighborhoods are so densely populated that emergency vehicles cannot pass through narrow alleyways. This combination of risk factors creates a situation whereby fires might break out in one home and spread rapidly across a whole community.

A second environmental risk we witness regularly in South Africa is the storage of paraffin. Shopkeepers store paraffin in bulk using badly maintained containers such as drums and above ground storage tanks. The absence of proper packaging can lead to contamination of paraffin; contaminated fuel causes air pollution and, in some cases, explosions and fires.

From a prevention perspective, local community leaders and government officials should interact with communities so that dwellings are built in orderly fashions that allow access to water hydrants. Fire retardant materials should be provided to communities for the construction of informal houses. Where local low-cost housing is being supplied by government entities, the homes should be built with a concrete cooking surface and with a flue, chimney and proper ventilation.

**Behavioral Approaches**

Attempts to change behavior surrounding safe paraffin use have achieved mixed results. This is not surprising: Psychologists have long recognized the challenge of changing human behavior, and recent innovations in health-related behavior change stress the fact that human behavior is very difficult to change. Convincing adults to store their paraffin on upper shelves so children will not reach them; to spend limited resources on proper storage containers for their paraffin; or to ensure tablecloths will not drop onto the top of paraffin stoves are not easy tasks.

The most frequently-used interventions for paraffin safety are public health marketing campaigns, conducted through use of posters and flyers in the neighborhood or through radio advertisements. These strategies achieve some change in paraffin-safety knowledge, but have failed to achieve concomitant changes in practice.^3-4^

Community-based educational interventions are among the most promising behavioral intervention to improve paraffin-related safety. Two recent studies suggest community-based education, conducted by respected local leaders, might help initiate some behavioral change in paraffin safety practice.^5-6^ In most parts of the world, low-income individuals derive great pleasure from socializing. Much of this socializing occurs with neighbors. Communities are densely populated, dwellings small and uncomfortable, and weather often hot. For this reason, neighbors naturally spend time conversing outdoors, and socialization cues in the neighborhood are likely to be prominent influences on behavior. That is, neighbors are likely to notice and mimic social cues from other neighbors. If one neighbor changes his or her behavior – and especially if that neighbor is socially respected – then others may also change their behavior. Stated in terms of social cognition theory, behavior change is likely to occur if the perceived norms in the community change.^7^ As people perceive safety-related behavior as normal, and notice their neighbors practicing safe behaviors, their own behavior change will follow.

Our own test of a community-based intervention designed to prevent paraffin-related injury was conducted in low-income communities near Cape Town, South Africa using a case-control design.^5^ With the primary objectives to change community norms and help citizens recognize their vulnerability to injury, we implemented a train-the-trainer model, whereby a professional educator trained local paraprofessionals, who then delivered the educational materials to the community. The local paraprofessionals were community leaders, and they went door-to-door in the community, visiting their neighbors, to educate people about paraffin safety. In doing so, they shared stories of their own family and home (change the perceived norms) and of neighbors who were victims of injury (increase perceived vulnerability to injury). In the end, we achieved significant paraffin-related safety behavior change in our intervention community, compared to a control community that received no intervention.

## Conclusions

Paraffin-related injury prevention must be a multifaceted, multidisciplinary, multisectoral and multicultural effort.^2^ Legislation is required in most nations of the world. Changes to the environments in which paraffin is manufactured, stored, and used are needed. Most challenging, we believe, is the need for behavioral change among paraffin users.

Overarching the challenge of paraffin safety is the need to balance culture specificity with global uniformity. As we have written in other domains of injury prevention,^8^ successful interventions must consider the cultural and educational specifics of the local environment, and must be tailored to the immediate needs. Concomitantly, the lessons learned in Cape Town are likely to be transferable, with cultural adjustments, to other locales. Dissemination of successes – and failures – is critical, so that we can share our knowledge across borders and together work to reduce the rates of paraffin-related injury.
